# Trends in cannabis-attributable hospitalizations and emergency department visits: data from the Canadian Substance Use Costs and Harms Study (2007–2020)

**DOI:** 10.24095/hpcdp.45.6.01

**Published:** 2025-06

**Authors:** Raadiya Malam, Rachael MacDonald-Spracklin, Emily Biggar, Adam Sherk, Anat Ziv, Robert Gabrys, Shea Wood, Matthew M. Young, Aisha Giwa, Chandni Sondagar, Jinhui Zhao, Pamela Kent, Tim Stockwell

**Affiliations:** 1 Canadian Centre on Substance Use and Addiction, Ottawa, Ontario, Canada; 2 Public Health Agency of Canada, Winnipeg, Manitoba, Canada; 3 Canadian Institute for Substance Use Research, University of Victoria, Victoria, British Columbia, Canada; 4 School of Public Health and Social Policy, University of Victoria, Victoria, British Columbia, Canada; 5 Greo Evidence Insights, Guelph, Ontario, Canada; 6 Carleton University, Ottawa, Ontario, Canada

**Keywords:** cannabis, hospitalizations, emergency department visits, psychotic disorder, acute intoxication, Canada

## Abstract

**Introduction:**

The prevalence of cannabis use continues to increase among certain populations in Canada. This study focussed on the increase in cannabis-attributable hospitalizations and emergency department (ED) visits from 2007 to 2020.

**Methods:**

To estimate the counts of hospitalizations and ED visits attributable to cannabis use, we acquired record-level hospital discharge data with ICD-10 diagnostic information for all fiscal years 2006/07 to 2020/21. Diagnostic information was used to associate each record to a health condition category for eight substances, including cannabis. The prevalence of cannabis use was estimated for each province or territory, calendar year, sex and age using national survey information. These estimates were used to adjust relative risk estimates derived from cannabis literature to calculate cannabis-attributable fractions, which were in turn used to estimate the proportion of hospitalizations and ED visits that were attributable to cannabis use.

**Results:**

Between 2007 and 2020, the overall rate of cannabis-attributable inpatient hospitalizations increased by 120%, from 6.4 in 2007 to 14.0 per 100000 in 2020. Cannabis-attributable ED visits increased by 113%, from 52.1 per 100 000 in 2007 to 111.0 per 100 000 in 2019, and then decreased by 12% in 2020. This study found that the increases in hospitalizations and ED visits were partly attributed to neuropsychiatric conditions, particularly hospitalizations due to psychotic disorders and ED visits due to acute intoxication among children and youth.

**Conclusion:**

Ongoing monitoring of cannabis-attributable harms is necessary to understand the harms related to use and the factors that influence the ways in which people use cannabis and seek care. Further research may distinguish the early effects of legalization trends from the early pandemic period data.

**Highlights**

- In Canada in 2020, cannabis was responsible for an estimated 5318 hospitalizations and 37 341 emergency department visits.
- The rate of cannabis-attributable hospitalizations and emergency department visits increased over the study period (2007–2020) by 120% and 88%, respectively, with notable increases among people with neuropsychiatric conditions and unintentional injuries.
- Rates of hospitalizations for cannabis- attributable psychotic disorders were highest among those aged 15 to 34 years. The crude rate among those aged 35 to 64 years increased by 38% between 2019 and 2020.

## Introduction

Cannabis continues to be one of the most-used psychoactive substances in Canada after alcohol.^1^ The prevalence of cannabis use has risen steadily over the past decade, with rates continuing to increase since the legalization of nonmedical cannabis in October 2018.^2^ From 2007 to 2020, past-year cannabis use among those aged 15 years and older increased from 11% to 18%.^3^ In 2022, past-year cannabis use among the general population (aged 16 years and older) was 27%, an increase from 25% in 2021.^4^ Overall, among those who used cannabis in the past year, 23% reported daily or almost daily use of cannabis in 2023, with findings showing that more males than females used cannabis daily (25% vs. 20%, respectively).^5^

Alongside this observed increase in the prevalence of cannabis use over the past several years, there is also some evidence of increases in cannabis-related harms.^6^ Cannabis-related harms can include injuries from motor vehicle collisions and other unintentional injuries, an increased risk of psychosis and cannabis use disorder as well as some conditions that arise during pregnancy, such as low birth weight.^7^,^8^ There is more literature on hospitalizations and emergency department (ED) visits due to cannabis-attributable harms, as cannabis has been the most common cause of substance use hospitalizations among youth in Canada since legalization.^9^,^10^

In addition, increases in age-specific rates of ED visits due to cannabis poisoning have been observed,^11^ as has an increased prevalence of injured drivers testing positive for tetrahydrocannabidiol (THC) since legalization.^12^ Other research has also explored the increase in cannabis use and associated harms before legalization.^6^,^13^ For example, Maloney-Hall et al. showed that hospitalizations for psychotic disorders due to cannabis use tripled from 2005 to 2015.^13^

In the present study, we built on this existing literature on cannabis use hospitalizations and ED visits to create a 14-year time series. 

This study was based on the ongoing Canadian Substance Use Costs and Harms (CSUCH) project, which is the only study in Canada to estimate costs and harms dating back to 2007 across a range of substances and outcomes.^3^ CSUCH estimates costs and harms for eight types of psychoactive substances, including cannabis. The study also estimates cannabis-attributable costs and harms across 20 indicators in the domains of health care, economic loss of production and criminal justice, and does so over the 14-year period from 2007 to 2020.^3^ During the study period (2007 to 2020), several significant cannabis policy events occurred, any of which may have influenced trends in cannabis-related harms. These include changes to medical cannabis regulations (the enactment of the *Marihuana for Medical Purposes Regulations* in 2013 and the *Access to Cannabis for Medical Purposes Regulations* in 2016), the Government of Canada’s 2016 announcement of its intent to legalize and regulate nonmedical cannabis, the enactment of the *Cannabis Act* in October 2018, and the onset of the COVID-19 pandemic in early 2020.

To further assess the trends over the 14-year period, the potential impact of legalization of nonmedical cannabis and the potential impact of the COVID-19 pandemic on cannabis-attributable harms, we examined cannabis-attributable hospitalizations and ED visits from 2007 to 2020 and trends from the early years after legalization (2019–2020) across several cannabis-related health conditions. 

## Methods

The CSUCH 2007–2020 methodology was originally based on the cost study by Rehm et al.^14^ and existing international guidelines and literature for which cost estimates of substance use were developed for other countries.^15^,^16^

***Data sources***

Record-level discharge data corresponding to overnight inpatient hospitalizations and ED visits were requested and received from the Canadian Institute for Health Information (CIHI) for health care fiscal years 2006/07 to 2020/21. These data were from CIHI’s Discharge Abstract Database (DAD)^17^ and National Ambulatory Care Reporting System (NACRS).^18^ As part of this request, we also received record-level costing information in the form of CIHI’s Case Mix Groups+/Resource Intensity Weights, Comprehensive Ambulatory Classification System (CMG+/RIW, CACS) and cost of a standard hospital stay (CSHS; by province/territory) variables, which allowed us to assign an approximated cost to each discharge record.^19^

Inpatient data are from all provinces and territories except Quebec, for all study years. The use of Quebec data requires special permission; this permission was not received in a timely manner and so data could not be included. Only two provinces (Ontario [for 2006/07–2020/21] and Alberta [for 2010/11–2020/21]) and one territory (Yukon, for years 2014/15–2020/21) reported emergency department data with diagnostic information (known as Level 3 data). NACRS Level 3 is required to group records into substance use–related health condition categories. 

Data on the prevalence of cannabis use came from the Canadian Substance Use Exposure Database (CanSUED).^3^,^20^ CanSUED was developed and is maintained for the CSUCH project and is based on estimates from the suite of national substance use surveys, which include the Canadian Alcohol and Drugs Survey (CADS); the Canadian Tobacco, Alcohol and Drugs Survey (CTADS); the Canadian Tobacco Use Monitoring Survey (CTUMS); and the Canadian Alcohol and Drug Use Monitoring Survey (CADUMS). The prevalence estimates were modelled by different age* (0–14, 15–34, 35–64, 65+ years) and sex (male, female) groups and by province/territory and year (2007–2020). 

***Identifying cannabis-related health conditions and estimating cannabis-attributable fractionsand cannabis-attributable hospitalizations and ED visits***

To identify health conditions and diseases causally related to cannabis use, ​we used Rehm et al.^14^ and preparatory material from the most recent Global Burden of Disease study estimates.^21^ Relative risk information corresponding to each identified partially-attributable health condition was from the literature (see Supplementary Table S1).^22^-^30^ The cannabis-related health conditions† identified were mental and behavioural disorders‡ due to the use of cannabinoids (F12), conditions arising during pregnancy due to cannabis use (O35.5, P04.4, P96.1), motor vehicle collisions (V1, Y85.0), unintentional injuries such as accidental poisoning (T40.7, X41–X44, Y11–Y14) and fires (X00–X09, Y26), intentional poisoning (T40.7, X61–X64) and assault/homicide (X85–Y09, Y87.1). 

The F12 category corresponding to mental and behaviouraldisorders due to the use of cannabinoids is wholly attributable to cannabis; that is, a condition of this type could not occur in the absence of cannabis use. Epidemiologically, it therefore has a cannabis-attributable fraction of 1.00. Other health conditions, however, are partially attributable to cannabis use (e.g. motor vehicle collisions, nonviolent crime), meaning cannabis use increases the risk of these conditions, but the condition can also occur in the absence of cannabis use. The proportion of each health condition, by year, province/territory, sex and age group, was estimated by calculating cannabis-attributable fractions (CAFs) using Formula 1:



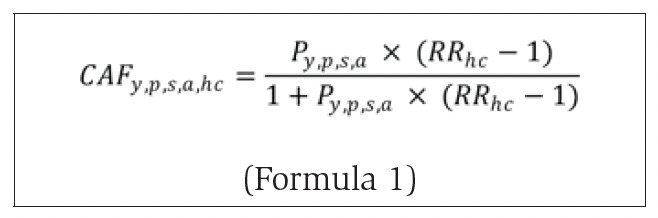



where *P*_y_,_p_,_s_,_a_ is the prevalence of past-year cannabis use, by each year, province/territory, sex and age group and *RR*_hc_ is the relative risk of each partially attributable cannabis-related health condition.

Cannabis-attributable events (hospitalizations and ED visits) were then estimated using the following methodology. First, discharges were enumerated into as many as one cannabis-related health condition using the ICD-10 code present as the most responsible diagnosis, except for a differential methodology for injuries and poisoning. Discharges relating to injuries and poisonings are also assigned an external cause code, which describes how the injury (e.g. a broken arm) came about (e.g. a motor vehicle collision). In this case, the external cause code is used to group the discharge into as many as one cannabis-related health condition. The CAFs calculated using Formula 1 are then applied by year, province/territory, sex, age group and health condition to arrive at an estimated number of cannabis-attributable hospitalizations and ED visits.

***Estimating cannabis-attributable rates and associated health care costs***

Population-based rates were calculated by dividing the count in each population subgroup by the corresponding population on 01 July of the year in question, as reported by Statistics Canada.^31^ Record-level cost estimates were generated by multiplying the CMG+/RIW (for inpatient) or CACS RIW (for ED visits) by the CSHS, as opposed to counting the record as 1.0. Cost calculations were then estimated by summing record-level cost estimates.

From 2016 to 2020, a focussed analysis was completed for health conditions in the F12 category–collectively, mental and behavioural disorders due to the use of cannabinoids. These F12 conditions were grouped into five subcategories, namely, acute intoxication (F12.0), dependence and withdrawal (F12.2, F12.3, F12.4), harmful use (F12.1), psychotic disorder (F12.5, F12.7) and all other (F12.6, F12.8, F12.9). Rates were calculated using the same population figures as described earlier.

***Emergency department imputation methods***

Reporting information for ED visits at the provincial/territorial level was incomplete. Only Ontario (for fiscal years 2006/07–2020/21), Alberta (2010/11–2020/21) and Yukon (2014/15–2020/21) reported complete diagnostic information (corresponding to NACRS Level 3). Therefore, these three jurisdictions were used in the primary ED cost analysis, and the costs and number of visits for all other provinces and territories were imputed from this basis.To impute the costs for all other provinces, summary tables for Ontario and Alberta were created with ED visit costs for province/territory, year, sex, age group and condition for each substance. These summary tables were rolled up across health conditions, resulting in a table with costs for each province/territory, year, sex and age group. 

With information from Alberta and Ontario from 2007 to 2020 and Yukon from 2015 to 2020, the ED costs for other provinces were imputed based on Formula 2:



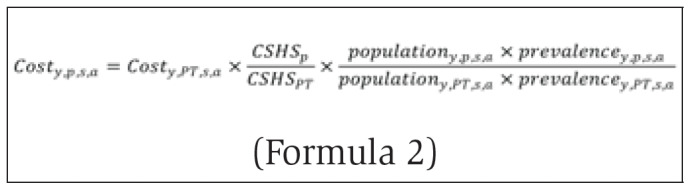



where *y* is year, *p* is the province/territory being imputed, *PT* is the province/territory with full information (Ontario, Alberta or Yukon) used to do the imputation, *s* is sex, *a* is age group and *CSHS* is the provincial-level cost of a standard hospital stay variable from the Canadian Institute for Health Information. The costs were adjusted by a “health care cost difference” factor. This factor was based on the ratio of the CSHS variables from Ontario and Alberta. 

***Analysis***

Hospitalization and ED visits data were presented in costs and crude rates (per 100 000) for the age groups 0 to 14 years, 15 to 34 years, 35 to 64 years and 65+ years, and grouped by male and female. Further, we calculated the percent change from 2019 (the first complete year of data after Canada legalized cannabis) to 2020 for cannabis-attributable hospitalizations for unintentional injuries to examine the trend during the first two years after legalization among males and females. Throughout this paper, we calculated the percent change from 2007 to 2020 in order to understand the general trends over the entire study period. Lastly, we examined hospitalizations and ED visits for mental and behavioural disorders due to cannabis, analyzing the F12 condition category from 2016 to 2020 (shown in the Results section). All analyses were performed in R statistical software version 4.3.3 (R Foundation for Statistical Computing, Vienna, AT) and Excel (Microsoft Corp., Redmond, WA, US).

*As part of the comprehensive national cost study (CSUCH), these age groups were selected to maintain consistency across 20 indicators. The underlying cost study also gathers data for several variables (i.e. province/territory, sex, age, substances, years and health conditions, where applicable). Due to disclosure issues for some datasets, further granularity among age groups would present challenges.†When discharged from Canadian hospitals, patients are assigned a code that indicates the main reason for their hospital stay. Different databases use different coding systems; however, the Discharge Abstract Database uses the Canadian modification of the International Statistical Classification of Diseases and Related Health Problems, 10th Revision (ICD-10-CA), which is reflected in our methodology.‡Conditions included in this diagnosis (ICD-10-CA code F12.0–12.9) were acute intoxication, harmful use, dependence syndrome, withdrawal state, withdrawal state with delirium, psychotic disorder, amnesic syndrome, residual and late-onset psychotic disorder, other mental and behavioural disorders and unspecified mental and behavioural disorder.

## Results

***Inpatient hospitalizations***

**Overall trends**

In 2020, cannabis was responsible for an estimated 5318 hospitalizations. The overall crude rate of cannabis-attributable inpatient hospitalizations increased by 120% between 2007 and 2020, with the majority of this increase occurring prior to legalization (2007–2016; Table 1). Examining the first years after legalization, hospitalizations increased from a rate of 12.8 per 100000 in 2019 to 14.0 per 100000 in 2020 (a 9% increase). The largest percentage increase in crude rates of cannabis-attributable hospitalizations over the study period occurred among youth: females under 15 years of age experienced a 247% increase and males under 15 experienced a 226% increase. Also, in the 15 to 34 age group, the difference between the rates for males and females increased the most compared to the other age groups.

**Table 1:** Overall crude rate (per 100 000) of cannabis-attributable hospitalizations, by sex and age, Canada, 2007 to 2020

Variable	2007	2008	2009	2010	2011	2012	2013	2014	2015	2016	2017	2018	2019	2020	% change 2007–2020
**Female **	4.1	4.2	3.7	4.4	5.0	5.2	6.2	6.3	7.6	8.2	8.3	9.0	8.8	9.5	134
0–14 y	4.1	4.3	4.5	5.9	7.6	7.9	9.3	9.7	10.9	11.8	11.1	13.7	11.6	14.1	247
15–34 y	6.5	6.9	5.7	6.7	7.5	7.4	10.0	9.6	13.0	13.9	15.1	14.8	14.8	15.9	146
35–64 y	3.0	3.1	2.7	3.0	3.2	3.5	3.7	4.1	4.6	5.0	4.8	5.4	5.5	6.4	111
65+ y	2.7	2.7	2.3	2.5	3.2	3.2	3.5	3.6	4.0	4.4	4.5	5.1	5.4	4.0	48
**Male **	8.7	8.6	8.3	9.8	11.5	12.0	12.7	13.9	14.5	16.5	16.9	16.8	16.9	18.5	113
0–14 y	5.6	6.2	6.3	7.9	10.6	11.3	12.6	14.0	13.9	15.6	18.1	15.3	15.7	18.2	226
15–34 y	16.8	16.5	15.5	18.7	21.5	22.6	23.9	26.1	27.2	30.9	30.6	31.6	30.4	33.0	97
35–64 y	6.2	6.1	5.9	6.6	7.5	7.7	8.1	8.7	9.6	11.2	11.3	11.5	12.3	13.9	123
65+ y	3.2	3.1	3.0	3.7	4.3	4.7	4.7	5.4	5.5	6.5	6.7	6.8	7.3	6.7	107
**Total **	6.4	6.4	6.0	7.1	8.2	8.6	9.4	10.1	11.0	12.3	12.6	12.9	12.8	14.0	120

**Data source: **Canadian Institute for Health Information Discharge Abstract Database, 2006/07 to 2020/21, and National Ambulatory Care Reporting System, 2006/07 to 2020/21. 

**Abbreviation:** y, years.

^a^ Excluding Quebec. 

**Trends by health condition category**

**Figure 1:**
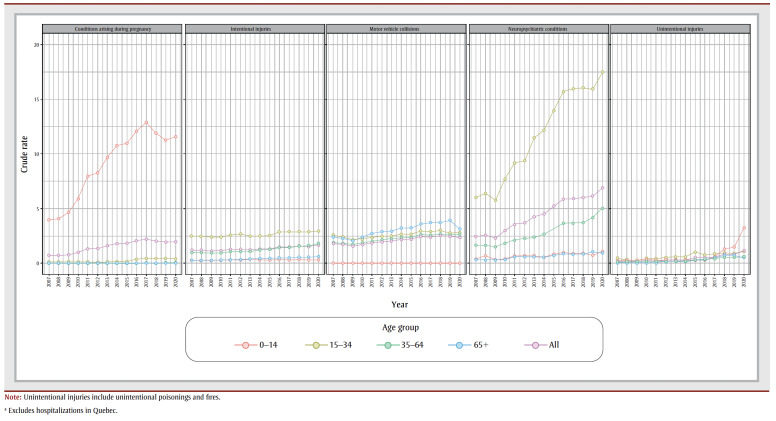
Crude rate (per 100 000) of cannabis-attributable hospitalizations, by health condition and age group, Canada, 2007 to 2020

Between the first two years after legalization (2019–2020), the largest increase in crude rates of hospitalizations was observed for unintentional injuries (41%). However, comparatively, between the two years before legalization (2016–2018), the crude rate of hospitalizations for unintentional injuries increased by 72% (see Supplementary Figure S1). Also, from 2019 to 2020, the increase among those aged 0 to 14 years was 124% (Figure 1), and while the crude rate was higher among males than females for most of the years, the rate of change of hospitalizations for unintentional injuries between 2019 and 2020 was higher among females (60%) than males (24%; Supplementary Table S2). 

Per person costs for neuropsychiatric conditions (mental and behavioural disorders due to cannabis) increased the most from 2007 to 2020 (160%), followed by per person costs for unintentional injuries (150%; Supplementary Figure S2). Neuropsychiatric conditions also accounted for an increasing proportion of all cannabis-attributable hospitalizations over the study period from 2007 to 2020 (Supplementary Table S3), and rates were highest among the 15 to 34 age group (Figure 1). The rate of hospitalizations for neuropsychiatric conditions remained relatively stable from 2016 to 2019, and increased between 2019 and 2020 (13%; Supplementary Table S3). From 2019 to 2020, the largest increase occurred among the 0 to 14 age group (51%), followed by the 35 to 64 age group (22%; Figure 1). Also, briefly analyzing the hospitalizations for cannabis-attributable motor vehicle collisions, we see that the rates peaked in 2018, representing a 37% increase from 2007 (Figure 1). This rate decreased slightly (6%) after 2018.

**Trends by F12 condition category for mental and behavioural disorder due to cannabis use**

*Age*

Further analyses of the neuropsychiatric conditions, in particular the F12 condition category (Table 2), showed that between 2019 and 2020, the crude rate of cannabis-attributable hospitalizations for psychotic disorder (F12.5) increased by 38%, from 2.0 to 2.8 per 100 000, among those aged 35 to 64 years. Over the same period, rates of hospitalizations for psychotic disorder among those aged 15 to 34 years also increased, by 21% from 8.7 to 10.6 per 100 000. In comparison, leading up to legalization, between 2016 and 2018, rates increased by 22% and 10% among those aged 35 to 64 years and 15 to 34 years, respectively. Moreover, cannabis-attributable hospitalizations for acute intoxication nearly tripled from 2019 to 2020 among those aged 0 to 14 years. Overall rates of dependence and withdrawal remained stable from 2019 to 2020; however, they decreased among those aged 15 to 34 (19%) and 35 to 64 years (16%).

**Table 2:** Crude rate of cannabis-attributable hospitalizations (per 100 000) by F12 condition category for mental and behavioural disorder due to cannabis use, by age, Canada, 2016 to 2020

F12 condition	2016	2017	2018	2019	2020
**Acute intoxication **	0.3	0.2	0.4	0.3	0.3
0–14 y	0.1	0.1	0.2	0.1	0.3
15–34 y	0.5	0.6	0.6	0.5	0.4
35–64 y	0.2	0.1	0.3	0.3	0.2
65+ y	0.3	0.1	0.4	0.4	0.3
**Dependence and withdrawal **	0.5	0.4	0.4	0.4	0.4
0–14 y	0.1	0.0	0.0	0.0	0.1
15–34 y	1.1	1.1	1.0	1.1	0.9
35–64 y	0.3	0.3	0.2	0.3	0.2
65+ y	0.1	0.1	0.0	0.1	0.1
**Harmful use **	1.1	1.2	1.1	1.1	1.0
0–14 y	0.3	0.5	0.3	0.2	0.3
15–34 y	3.2	3.3	3.1	3.1	2.5
35–64 y	0.5	0.4	0.5	0.6	0.6
65+ y	0.0	0.1	0.1	0.1	0.0
**Psychotic disorder **	2.6	2.6	2.8	3.2	4.0
0–14 y	0.3	0.2	0.2	0.2	0.3
15–34 y	7.3	7.1	8.1	8.7	10.6
35–64 y	1.3	1.6	1.6	2.0	2.8
65+ y	0.2	0.2	0.0	0.2	0.3
**All other **	0.6	0.7	0.7	0.7	0.7
0–14 y	0.2	0.1	0.2	0.1	0.1
15–34 y	1.7	2.0	2.2	1.8	2.0
35–64 y	0.3	0.3	0.3	0.4	0.5
65+ y	0.1	0.0	0.0	0.1	0.0
**Total **	5.0	5.1	5.4	5.7	6.4

**Abbreviation**: y, years. 

^a^ International Statistical Classification of Diseases and Related Health Problems, 10th Revision, Canada (ICD-10-CA) code category.

^b^ Excludes hospitalizations in Quebec.

*Sex*

The crude rate for cannabis-attributable hospitalizations due to psychotic disorder for males was higher compared to females (Supplementary Table S4), both leading up to legalization (2016–2018) and during the first two years after legalization (2019–2020); however, females had a greater percentage increase from 2019 to 2020 (34%) compared to males (22%).

***Emergency department visits***

**Overall trends**

In 2020, cannabis was responsible for an estimated 37341 ED visits. From 2007 to 2019, per person costs for ED visits for cannabis increased by 95%, subsequently decreasing slightly in 2020 (Supplementary Table S5). Similarly, the crude rate of cannabis-attributable ED visits increased from 2007 to 2019 by 113%, and then decreased between 2019 and 2020 by 12%. 

**Trends by age group and sex**

**Figure 2:**
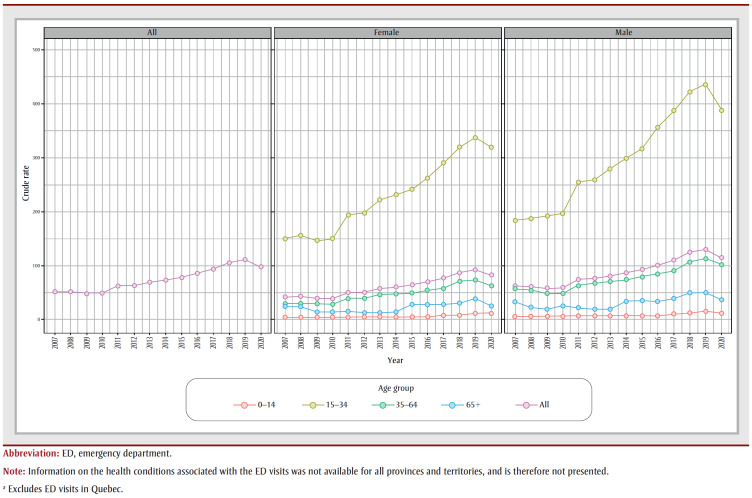
Crude rate (per 100 000) of cannabis-attributable ED visits, by age group and sex, Canada, 2007 to 2020

Rates of ED visits due to cannabis use peaked in 2019 among all age groups and subsequently decreased in 2020 (Figure 2). Over the study period (2007–2020), the rates of ED visits were highest for both males and females aged 15 to 34 years. Also, the 0 to 14 age group had the greatest percentage increase (136%) followed by the 15 to 34 (112%), 35 to 64 (91%) and 65 and older (9%) groups. During the first two years after legalization (2019–2020), all age groups experienced a decline in cannabis-attributable ED visits. Additionally, cannabis-attributable ED visits were continuously higher among males than females. From 2007, rates of ED visits among males and females increased by 108% and 120%, respectively, until 2019 and then decreased by 12% and 11% in 2020 (Figure 2). 

**Trends by F12 condition category for mental and behavioural disorder due to cannabis use**

*Age*

In 2020, among those aged 0 to 14, acute intoxications (F12.0) accounted for most of the ED visits for mental and behavioural disorder due to cannabis use (48%), followed by harmful use (F12.1) (43%; Table 3). Further, in 2020, 42% of all ED visits for mental and behavioural disorders among those aged 15 to 34 years were for harmful use and 27% were for acute intoxication. From 2016 to 2018, acute intoxications among all age groups increased by 135%. 

**Table 3:** Crude rate of cannabis-attributable ED visits (per 100 000) by F12 condition category for mental and behavioural disorder due to cannabis use, by age, Canada, 2016 to 2020

F12 condition	2016	2017	2018	2019	2020
**Acute intoxication **	4.5	6.7	10.6	12.7	11.1
0–14 y	1.0	2.0	2.8	4.4	2.9
15–34 y	12.2	17.6	26.1	28.2	24.9
35–64 y	2.2	3.4	6.4	9.2	8.1
65+ y	1.0	1.6	3.0	3.8	3.5
**Dependence and withdrawal **	1.7	2.0	2.0	2.3	2.6
0–14 y	0.2	0.3	0.1	0.2	0.2
15–34 y	4.9	5.5	6.0	6.6	7.6
35–64 y	0.9	1.0	0.9	1.0	1.3
65+ y	0.0	0.2	0.1	0.2	0.0
**Harmful use **	8.1	10.6	12.6	14.7	14.7
0–14 y	1.8	2.7	2.9	3.7	2.6
15–34 y	23.2	30.5	34.7	37.5	38.7
35–64 y	3.5	4.2	6.1	8.8	8.8
65+ y	0.6	0.9	1.4	1.9	1.6
**Psychotic disorder **	2.4	3.3	3.9	4.5	5.4
0–14 y	0.1	0.1	0.1	0.2	0.1
15–34 y	7.5	9.8	11.5	12.9	15.5
35–64 y	0.9	1.4	1.9	2.2	3.0
65+ y	0.1	0.1	0.0	0.2	0.1
**All other **	1.1	1.3	1.6	2.1	1.9
0–14 y	0.2	0.2	0.2	0.3	0.3
15–34 y	3.4	3.7	4.8	5.5	5.3
35–64 y	0.4	0.5	0.7	1.2	1.0
65+ y	0.1	0.0	0.2	0.3	0.2
**Total **	17.9	23.8	30.8	36.2	35.8

**Abbreviations**: ED, emergency department; y, years. 

^a^ International Statistical Classification of Diseases and Related Health Problems, 10th Revision, Canada (ICD-10-CA) code category. 

^b^ Excludes ED visits in Quebec. 

Between 2019 and 2020, the crude rate for psychotic disorder (F12.5) among those aged 15 to 34 years increased the most—by 20%, from 12.9 to 15.5 per 100 000. Over the same period, across the 15 to 34 and 35 to 64 age groups, ED visits for dependence syndrome and withdrawal (F12.2, F12.3, F12.4) increased by 15% and 32%, respectively. 

*Sex*

In 2020, the crude rate for cannabis-attributable ED visits for mental and behavioural disorders among both males and females (Supplementary Table S6) were highest for harmful use, followed by acute intoxication. Between 2019 and 2020, the crude rate for harmful use among females increased the most, from 10.8 to 11.8 per 100 000 (10%), and decreased for males from 18.7 to 17.7 per 100 000 (5%). 

## Discussion

Overall, across all age groups and health conditions, inpatient hospitalizations and ED visits increased from 2007 to 2020. Legalization of nonmedical cannabis in 2018 and the COVID-19 pandemic in 2020 may have influenced these trends, particularly impacting hospitalizations due to unintentional injuries and neuropsychiatric conditions; however, cannabis use and related harms have been increasing over time.^6^ These findings are consistent with increasing research showing a link between frequent and long-term use of cannabis and psychotic and other mental health disorders.^13^,^32^

***Inpatient hospitalization trends by health condition, age and sex***

The crude rate of inpatient hospitalizations due to cannabis use increased by 120% between 2007 and 2020 and 9% during the first two years after legalization of nonmedical cannabis in late 2018 (2019–2020). Increased hospitalizations for neuropsychiatric conditions, cannabis-attributable motor vehicle collisions and unintentional injuries (including poisonings) contributed to this trend. Per person costs of hospitalizations for neuropsychiatric conditions increased the most over the 14 years, followed by per person costs for unintentional injuries, while the crude rate of hospitalizations for unintentional injuries showed the largest increase (more than 5 times) over the study period (2007–2020). In the two years leading up to legalization (2016–2018) the rate of hospitalization for unintentional injuries increased by 72%. From 2019 to 2020, these rates continued to increase, though to a lesser extent (41%) than prior to legalization. Those aged 0 to 14 and females had the largest increases in crude rates of unintentional injury hospitalizations after 2018 (124% and 60%, respectively). 

It is important to note that for the first year of legalization (October 2018 to October 2019), cannabis edibles were not yet legal for sale in Canada. Research suggests that edible products (e.g. gummies, candies, chocolate, baked goods) have been associated with more accidental poisonings than other forms of cannabis.^33^ While this report presents the national trend, other studies have found that the rates of hospitalizations for cannabis poisoning among children were higher in jurisdictions that did not restrict certain types of edible products, specifically products that are appealing to children and youth.^34^,^35^ Continued education concerning the safe storage of edible products and future monitoring both of trends since edible products became widely available on the legal market and of products considered to be appealing to children and youth will be necessary to determine whether further policy on the sale, packaging, storage or labelling of such products is needed to prevent cannabis-related harms, such as poisoning, especially among those aged 0 to 14 years.^36^-^38^

Most cannabis-attributable hospitalizations across the study period were for neuropsychiatric conditions, and these continued to increase after 2018, particularly among the 0 to 14 age group. These conditions include a range of diagnoses, including psychoses. Hospitalizations for psychotic disorder among those aged 35 to 64 increased by 38% from 2019 to 2020. Other studies have also reported an increase in hospitalizations and ED visits for cannabis-related psychosis after legalization.^10^,^39^ This increase may be in part due to improved and broadened health care provider knowledge on cannabis use and related conditions in Canada.^40^ However, other studies have found inconclusive evidence of an increase in psychiatric presentations after legalization.^40^

There is evidence that higher levels of cannabis use increases the risk of psychotic outcomes.^41^ Higher potency cannabis has been linked to an increased risk of psychosis^42^ and some studies show that cannabis use frequency is associated with increased risk of neuropsychiatric disorders, particularly among young people.^43^-^45^ Research shows that there are gender-specific risks and harms associated with cannabis use, specifically with frequency and patterns of use.^46^ For example, young men used more frequently, in larger amounts, and were more likely to use alone, increasing their risk of dependency and mental illnesses compared to that of young women.^46^

Accordingly, close monitoring of trends related to cannabis-attributable neuropsychiatric conditions and cannabis indicators (including frequency of use, potency of products used and overall level of cannabis exposure) will be needed to further establish the patterns of potential harm. The current literature highlights the need for making access to mental health and addiction services more equitable, and for public education,^47^ especially for youth and young adults, as current education is inadequate.^48^

***ED visit trends by health condition, age and sex***

The overall per person costs of cannabis-attributable ED visits increased by 95% from 2007 to 2019, and slightly decreased (by 2%) between 2019 and 2020.^3^ The overall crude rate of ED visits also increased (113%) between 2007 and 2019 and decreased (12%) from 2019 to 2020. These harms increased the most in the 0 to 14 and 15 to 34 age groups from 2007 to legalization in 2018. Myran and colleagues also found that cannabis-attributable ED visits increased prior to legalization in late 2018.^35^

Moreover, in 2020, almost half (48%) of cannabis-attributable ED visits due to mental and behavioural disorders among the 0 to 14 age group were for acute intoxications. Though research has found that following legalization, there was no difference in the overall rate of ED visits due to acute intoxication, visits among adults aged 18 to 29 increased by more than 50%.^49^ We also found that ED visits for dependence syndrome and withdrawal increased from 2019 to 2020, among those aged 15 to 34 and 35 to 64 years. Additionally, researchers have concluded that overall, harmful use, acute intoxication and dependency were the most common causes for cannabis-involved traffic ED visits from 2010 to 2021.^50^

In general, rates of ED visits in all age groups were likely impacted by the pandemic-related reduction in health care services usage, such as ED utilization, and disrupted daily patterns of life.^3^,^51^,^52^ Research finds that evolving stringency of cannabis retail restrictions in some provinces also impacted the trends observed within the pandemic period.^35^ Therefore, to determine post-legalization trends for ED visits due to cannabis use, continued monitoring is required. Future research may explore how jurisdictional cannabis regulations have impacted cannabis-attributable harms in the pandemic-recovery period.

***Strengths and limitations***

One main strength of our study is that we estimated substance use harms that are wholly and partially attributable to cannabis use from 2007 to 2020, allowing for data analysis over time. However, there are some limitations. The Ontario Mental Health Recording System (OMHRS) was excluded from the calculation of inpatient hospitalizations due to the incompliance of the database with the ICD-10 classification system. Thus, for Ontario and Manitoba, there is likely an underestimation of hospitalization counts and costs. 

An additional limitation of this study, and of the underlying cost study, is the large amount of data imputation that is required for the ED visits analysis. NACRS Level 3 data were only available for three provinces/territories (Ontario for fiscal years 2006/07–2020/21, Alberta for fiscal years 2010/11–2020/21 and Yukon for 2015–2020). Therefore, ED visits costs and counts were imputed for the other provinces using the standard provincial groups, to report at a national level. Also, estimates were available only up until 2020. This makes it difficult to tease apart the early effects of legalization from those of the pandemic. Other evidence demonstrates that hospitalizations and ED visits for cannabis poisonings and mental and behavioural disorders continued to increase between 2020 and 2021.^53^ In addition to an increase in the rate of cannabis-attributable ED visits and hospitalizations among children, other studies have found an increased severity of ED visits, especially among children aged 12 years and younger.^35^,^54^-^56^

## Conclusion

This paper presents indicators of cannabis-attributable harms and associated costs over a 14-year period. Our findings show an increasing trend of cannabis-attributable hospitalizations and ED visits for neuropsychiatric conditions and poisonings, particularly among children and youth. Therefore, comprehensive monitoring of cannabis harms is needed, as well as adequate services and education to ensure the health and safety of those at risk. 

## Acknowledgements

The authors would like to thank and acknowledge John Dorocicz for his contributions, since 2016, to the methodology and analysis of this study and throughout the entirety of the Canadian Substance Use Costs and Harms study. The authors would also like to thank and acknowledge Cathleen de Groot for her technical help and information specialist expertise with the manuscript. 

## Funding

This work was made possible by financial support from Health Canada. 

## Conflicts of interest

The authors have no conflicts of interest to declare.

## Authors’ contributions and statement

EB, RMS: conceptualization.

RM, AS, RMS: formal analysis.

PK, TS, EB, AG, AS, RM, JZ, AZ (Canadian Substance Use Costs and Harms Working Group): methodology (underlying study).

AS: methodology (current study). 

RM: project administration.

RM: visualization.

RM, RMS: writing—original draft.

RM, AS, RG, CS, SW, MY: writing—review and editing.

The content and views expressed in this article are those of the authors and do not necessarily reflect those of the Government of Canada.

## References

[B01] (2020). Cannabis. Canadian Centre on Substance Use and Addiction.

[B02] Research to insights: cannabis in Canada [Internet]. Statistics Canada.

[B03] Canadian substance use costs and harms 2007–2020 [Internet]. Canadian Centre on Substance Use and Addiction.

[B04] Canadian Cannabis Survey 2022: summary [Internet]. Government of Canada.

[B05] Canadian Cannabis Survey 2023: summary [Internet]. Government of Canada.

[B06] Imtiaz S, Nigatu YT, Ali F, et al (2023). Cannabis legalization and cannabis use, daily cannabis use and cannabis-related problems among adults in Ontario, Canada (2001-2019). Drug Alcohol Depend.

[B07] Fischer B, Hall W, Fidalgo TM, et al (2023). Recommendations for reducing the risk of cannabis use-related adverse psychosis outcomes: a public mental health-oriented evidence review. J Dual Diagn.

[B08] Memedovich KA, Dowsett LE, Spackman E, Noseworthy T, Clement F (2018). The adverse health effects and harms related to marijuana use: an overview review. CMAJ Open.

[B09] Fewer young people being hospitalized for substance use, but overall rates still higher than before the pandemic [Internet]. CIHI.

[B10] Myran DT, Gaudreault A, Konikoff L, Talarico R, Pacula R (2023). Changes in cannabis-attributable hospitalizations following nonmedical cannabis legalization in Canada. JAMA Netw Open.

[B11] Varin M, Champagne A, Venugopal J, et al (2023). Trends in cannabis-related emergency department visits and hospitalizations among children aged 0–11 years in Canada from 2015 to 2021: spotlight on cannabis edibles. BMC Public Health.

[B12] Brubacher JR, Chan H, Erdelyi S, Staples JA, Asbridge M, Mann RE (2022). Cannabis legalization and detection of tetrahydrocannabinol in injured drivers. N Engl J Med.

[B13] Maloney-Hall B, Wallingford SC, Konefal S, Young MM (2020). Psychotic disorder and cannabis use: Canadian hospitalization trends, 2006-2015. Maloney-Hall B, Wallingford SC, Konefal S, Young MM.

[B14] Rehm J, Baliunas D, Brochu S, et al (2006). The costs of substance abuse in Canada 2002. Canadian Centre on Substance Abuse.

[B15] Collins DJ, Lapsley HM (2008). The costs of tobacco, alcohol and illicit drug abuse to Australian society in 2004/05. Australian Government Department of Health and Ageing.

[B16] Single E, Collins D, Easton B, et al (2003). International guidelines for estimating the costs of substance abuse. World Health Organization.

[B17] (2022). Discharge Abstract Database, 2006/07 to 2020/21. CIHI.

[B18] (2022). National Ambulatory Care Reporting System (NACRS), 2006/07 to 2020/2021. CIHI.

[B19] (2022). Cost of a standard hospital stay, 2020/21 Methodology. CIHI.

[B20] Zhao J, Stockwell T, Sorge J, et al Development of the Canadian Substance Use Exposure Database (CanSUED): Modeling the prevalence of substance use in Canadian jurisdictions, 2006 to 2017. Development of the Canadian Substance Use Exposure Database (CanSUED): Modeling the prevalence of substance use in Canadian jurisdictions, 2006 to 2017. Research Square [Preprint].

[B21] Degenhardt L, Charlson F, Stanaway J, et al, hepatitis C (2016). Estimating the burden of disease attributable to injecting drug use as a risk factor for HIV, hepatitis C, and hepatitis B: findings from the Global Burden of Disease Study 2013. Estimating the burden of disease attributable to injecting drug use as a risk factor for HIV, hepatitis C, and hepatitis B: findings from the Global Burden of Disease Study 2013. Lancet Infect Dis.

[B22] Brown SW, Vanlaar WG, Robertson RD (2017). The alcohol and drug-crash problem in Canada: 2014 report. Canadian Council of Motor Transport Administrators.

[B23] Brown SW, Vanlaar WG, Robertson RD (2017). The alcohol and drug-crash problem in Canada: 2013 report. Canadian Council of Motor Transport Administrators.

[B24] Brown SW, Vanlaar WG, Robertson RD (2015). The alcohol and drug-crash problem in Canada: 2012 report. Canadian Council of Motor Transport Administrators.

[B25] (2013). Alcohol-crash problem in Canada: 2010. Canadian Council of Motor Transport Administrators.

[B26] (2010). Alcohol-crash problem in Canada: 2007. Canadian Council of Motor Transport Administrators.

[B27] (2011). Alcohol-crash problem in Canada: 2009. Canadian Council of Motor Transport Administrators.

[B28] (2010). Alcohol-crash problem in Canada: 2008. Canadian Council of Motor Transport Administrators.

[B29] Evarts B (2019). Fire loss in the United States during 2018. National Fire Protection Association.

[B30] (2014). The health consequences of smoking—50 years of progress: a report of the Surgeon General. Centers for Disease Control and Prevention (US).

[B31] Table 17-10-0005- 01: Population estimates on July 1, by age and gender [Internet]. Statistics Canada.

[B32] Myran DT, Harrison LD, Pugliese M, et al (2023). Transition to schizophrenia spectrum disorder following emergency department visits due to substance use with and without psychosis. JAMA Psychiatry.

[B33] Coret A, Rowan-Legg A (2022). Unintentional cannabis exposures in children pre-and post-legalization: a retrospective review from a Canadian paediatric hospital. Paediatr Child Health.

[B34] Myran DT, Tanuseputro P, Auger N, Konikoff L, Talarico R, Finkelstein Y (2023). Pediatric hospitalizations for unintentional cannabis poisonings and all-cause poisonings associated with edible cannabis product legalization and sales in Canada. JAMA Health Forum.

[B35] Myran DT, Pugliese M, Tanuseputro P, Cantor N, Rhodes E, Taljaard M (2022). The association between recreational cannabis legalization, commercialization and cannabis-attributable emergency department visits in Ontario, Canada: an interrupted time-series analysis. Addiction.

[B36] Hammond D (2021). Communicating THC levels and “dose” to consumers: implications for product labelling and packaging of cannabis products in regulated markets. Int J Drug Policy.

[B37] Matheson J, Foll B (2020). Cannabis legalization and acute harm from high potency cannabis products: a narrative review and recommendations for public health. Front Psychiatry.

[B38] Myran DT, Tanuseputro P, Auger N, Konikoff L, Talarico R, Finkelstein Y (2022). Edible cannabis legalization and unintentional poisonings in children. N Engl J Med.

[B39] Myran DT, Pugliese M, Roberts RL, et al (2023). Association between non-medical cannabis legalization and emergency department visits for cannabis-induced psychosis. Mol Psychiatry.

[B40] Hall W, Dawson D, Leung J (2023). The implementation and public health impacts of cannabis legalization in Canada: a systematic review. Addiction.

[B41] Marconi A, Forti M, Lewis CM, Murray RM, Vassos E (2016). Meta-analysis of the association between the level of cannabis use and risk of psychosis. Schizophr Bull.

[B42] Petrilli K, Ofori S, Hines L, Taylor G, Adams S, Freeman TP (2022). Association of cannabis potency with mental ill health and addiction: a systematic review. Lancet Psychiatry.

[B43] Freeman TP, Winstock AR (2015). Examining the profile of high-potency cannabis and its association with severity of cannabis dependence. Psychol Med.

[B44] Robinson T, Ali MU, Easterbrook B, et al (2022). Identifying risk-thresholds for the association between frequency of cannabis use and development of cannabis use disorder: a systematic review and meta-analysis. Drug Alcohol Depend.

[B45] Robinson T, Ali MU, Easterbrook B, Hall W, Jutras-Aswad D, Fischer B (2023). Risk-thresholds for the association between frequency of cannabis use and the development of psychosis: a systematic review and meta-analysis. Psychol Med.

[B46] Goodman A (2021). Differences in cannabis perceptions among Canadian adolescent boys and girls. Canadian Centre on Substance Use and Addiction.

[B47] Kourgiantakis T, Lee E, Kosar AK, et al (2023). Youth cannabis use in Canada post-legalization: service providers’ perceptions, practices, and recommendations. Subst Abuse Treat Prev Policy.

[B48] (2023). A public health perspective on cannabis legalization and regulation in Canada. Canadian Centre on Substance Use and Addiction.

[B49] Baraniecki R, Panchal P, Malhotra DD, Aliferis A, Zia Z (2021). Acute cannabis intoxication in the emergency department: the effect of legalization. BMC Emerg Med.

[B50] Myran DT, Gaudreault A, Pugliese M, Manuel DG, Tanuseputro P (2023). Cannabis-involved traffic injury emergency department visits after cannabis legalization and commercialization. JAMA Netw Open.

[B51] Finkelstein Y, Maguire B, Zemek R, et al (2021). Effect of the COVID-19 pandemic on patient volumes, acuity, and outcomes in pediatric emergency departments: a nationwide study. Pediatr Emerg Care.

[B52] Kim S, Rajack N, Mondoux SE, Tardelli VS, Kolla NJ, Foll B (2023). The COVID-19 impact and characterization on substance use-related emergency department visits for adolescents and young adults in Canada: practical implications. J Eval Clin Pract.

[B53] Unintended consequences of COVID-19: impact on harms caused by substance use, self-harm and accidental falls [Internet]. CIHI.

[B54] Callaghan RC, Sanches M, Heiden J, Kish SJ (2023). Impact of Canada’s cannabis legalisation on youth emergency department visits for cannabis-related disorders and poisoning in Ontario and Alberta, 2015-2019. Callaghan RC, Sanches M, Vander Heiden J, Kish SJ.

[B55] Cohen N, Blanco L, Davis A, et al (2022). Pediatric cannabis intoxication trends in the pre and post-legalization era. Pediatric cannabis intoxication trends in the pre and post-legalization era. Clin Toxicol (Phila).

[B56] Yeung ME, Weaver CG, Hartmann R, Haines-Saah R, Lang E (2021). Emergency department pediatric visits in Alberta for cannabis after legalization. Pediatrics.

